# A Case Report on Tricho-Hepato-Enteric Syndrome: The SKIC3 Gene in Focus

**DOI:** 10.7759/cureus.58015

**Published:** 2024-04-11

**Authors:** Thabet Zidan, Ameer Awashra, Ahmad Nouri, Layan Abu Alya

**Affiliations:** 1 Faculty of Medicine and Health Sciences, An-Najah National University, Nablus, PSE; 2 General Practice, Palestine Medical Complex, Ramallah, PSE

**Keywords:** genetic testing, failure to thrive, congenital diarrhea, syndromic diarrhea, tricho-hepato-enteric syndrome

## Abstract

Tricho-hepato-enteric syndrome (THES), also known as syndromic diarrhea, is a rare genetic disorder that causes intractable diarrhea, hair anomalies, facial dysmorphism, and liver abnormalities. Herein, we report the case of an eight-month-old male who was referred to our hospital due to symptoms of diarrhea, vomiting, and insufficient weight gain. The child was born via cesarean section following an uncomplicated pregnancy, with no history of admission to the neonatal intensive care unit (NICU). Since birth, the patient has been experiencing diarrhea and inadequate weight gain, necessitating multiple hospital admissions. Upon evaluation, genetic testing confirmed the diagnosis of THES. The management strategy included a variety of nutritional interventions and supportive care measures. Currently, the patient is in the pediatric intensive care unit (PICU), receiving total parenteral nutrition (TPN) and continuous supportive care. This case underscores the complexity of diagnosing and managing THES, highlighting the need for comprehensive care and close monitoring of the patient's condition.

## Introduction

Tricho-hepato-enteric syndrome (THES), also referred to as syndromic diarrhea, is an exceptionally rare autosomal recessive genetic disorder with an estimated incidence of one in 1,000,000 individuals. Its rarity and complex clinical presentation pose significant challenges to diagnosis and management. First identified by Stankler et al. in 1982 and further defined by Girault et al. in 1994, THES is characterized by a spectrum of symptoms, including intractable diarrhea starting in the neonatal period, facial dysmorphism, woolly hair, immunological dysfunction, mild intellectual disability, and intrauterine growth restriction, along with other abnormalities affecting the skin, liver, and heart [1,2].

THES is the first mendelian disease linked to a cytoplasmic exosome anomaly, and it typically arises from mutations in either of two specific genes: superkiller viralicidic activity 2 (*SKIV2L*) or tetratricopeptide (TPR) repeat domain-containing protein 37 (*TTC37*). Both *SKIV2L* and *TTC37* encode proteins integral to a superkiller complex, which plays a crucial role in the mRNA cytoplasmic degradation of the RNA exosome [1,2].

Despite advancements, THES still carries a significant mortality rate, with approximately 2-3 deaths per 12 patients. The predominant complications associated with THES encompass liver pathology and susceptibility to infections. The inevitable failure to thrive that is caused by early-onset intractable diarrhea warrants parenteral nutrition to improve growth. Immunoglobulin supplementation and liver graft emerge as viable strategies for infection prevention and severe liver disease cases [1,3].

This case presents an eight-month-old male who has complained of diarrhea since birth and has had multiple admissions to the hospital. The whole exome sequence (WES) analysis ultimately led to his THES diagnosis, and he is currently receiving treatment in the pediatric intensive care unit (PICU).

## Case presentation

An eight-month-old male infant was admitted to our medical facility, presenting with symptoms of vomiting and diarrhea. The child was born via cesarean section at 38 weeks of gestation due to placental insufficiency, with a birth weight of 2.4 kg. The pregnancy proceeded without complications, and the infant did not need admission to the neonatal intensive care unit (NICU). It is noteworthy that the parents were not related, and the patient has a healthy three-year-old older sister.

The patient had been experiencing significant diarrhea since birth. Initially, he was breastfed for 1 month, after which his diet was switched to a cow-milk-based formula. Episodes of vomiting accompanied this dietary change. Due to these symptoms, he was admitted to a different hospital with the suspicion of a cow milk protein allergy. As a result, his diet was switched to a soy-based formula, but with no improvement. 

At the age of 2 months, dehydration necessitated the patient’s admission to another hospital due to diarrhea associated with mucus and vomiting of normal gastric content. Furthermore, the patient continued to exhibit poor weight gain: his weight was 2.4 kg at birth (below the 3^rd^ percentile), 3.1 kg at 1 month (still below the 3^rd^ percentile), and 3.4 kg at 2 months of age (still below the 3^rd^ percentile). His length was 55 cm (between the 3^rd^ and 15^th^ percentiles), whereas his head circumference was 38 cm (above the 15^th^ percentile).

The patient’s vital signs were recorded as follows: a blood pressure of 85/54 mmHg, a heart rate of 137 beats per minute, an oxygen saturation level of 98%, and an axillary temperature of 36.7°C. The patient appeared well and active on physical examination but showed signs of moderate dehydration as evidenced by dry mucous membranes, the absence of tears, and depressed fontanelles. 

The patient's initial laboratory test results are shown in Table 1. He was initiated on a regimen of supplemental pancreatic enzymes and an extensively hydrolyzed, hypoallergenic formula. However, the patient’s condition did not improve, leading to his referral to a different hospital for further evaluation of malabsorption.

**Table 1:** Laboratory values upon admission to the pediatric ward at the age of 2 months

Blood test	Result	Reference range	Unit
Complete Blood Count (CBC)			
Hemoglobin (HGB)	9.0	9.5-14.5	g/dL
Hematocrit (HCT)	27.5	31-41	%
Red blood cells (RBC)	3.05	3.9-5.3	10^6^/uL
Mean cell hemoglobin concentration (MCHC)	32.7	31-35	g/dL
Mean cell hemoglobin (MCH)	29.5	27-31.2	pg
Mean cell volume (MCV)	90.2	80-97	fL
Platelets Count (PLT)	394	150-450	10^3^/uL
White blood cells (WBC)	7.2	4.6-11	10^3^/uL
Neutrophils	2.4	1.7-7.7	10^3^/uL
Lymphocytes	4.2	0.7-4.8	10^3^/uL
Monocytes	0.6	0.0-0.9	10^3^/uL
General Chemistry			
Sodium	134	135-145	mEq/L
Potassium	3.8	3.5-5.3	mEq/L
Calcium	7.88	8.6-10	mg/dL
Magnesium	1.87	1.6-2.6	mg/dL
Blood Urea Nitrogen (BUN)	7.22	6-20	mg/dL
Creatinine	0.30	0.7-1.2	mg/dL
Random blood sugar (RBS)	82	74-110	mg/dL
Hepatic			
Aspartate aminotransferase AST (GOT)	29	0-50	U/L
Alanine transaminase ALT (GPT)	14	0-41	U/L
Protein, total	3.8	6.6-8.7	g/dL
Albumin	2.63	3.8-5.4	g/dL
Endocrine (Thyroid)			
Thyroid-stimulating hormone (TSH)	5.558	0.35-4.94	µU/mL
Free thyroxine (T4 free)	0.80	0.7-1.48	ng/dL
Arterial Blood Gases (Room Air)			
pH	7.289	7.35–7.45	-
pCO_2_	31.5	35–45	mmHg
HCO_3_	14.8	22-26	mEq/L
Others			
Lactate dehydrogenase	21.58	4.5-19.8	U/L
C-reactive protein (CRP)	3.2	0-0.8	mg/dL

Following assessment for malabsorption, the diagnosis of THES was established through WES analysis. Genetic testing identified a homozygous variant (c.3070delC) (p.Pro1357fs*10) in the SKIC3 gene located on chromosome 5, along with an incidental mutation (a hemizygous variant: c.202G>A, p.Val68Me) in the G6PD gene. Comprehensive results of WES are presented in Table 2. Immunological evaluation revealed IgG levels of 101 mg/dL, IgM levels less than 18 mg/dL, and IgA levels less than 26 mg/dL.

**Table 2:** Single nucleotide variant (SNV) analysis results.

Gene	SKIC3	G6PD
Transcript	NM_014639.4	NM_001360016.2
Position	5:94818318	X:153764217
Nucleotide	c.4070delC	c.202G>A
Amino Acid	p.Pro1357fs*10	p.Val68Met
GnomAD	–	0.009061
dbSNP	rs1582849807	rs1050828
Zygosity	Homozygous	Hemizygous
ACMG classification	Likely Pathogenic	Pathogenic

The patient was discharged with a treatment regimen that included metronidazole, iron, vitamin D drops, an extensively hydrolyzed hypoallergenic formula, and monthly intravenous immunoglobulin (IVIG). However, despite these interventions, the patient continued to suffer from diarrhea and insufficient weight gain. At four months of age, his weight was 4 kg (falling below the third percentile), his length was 57 cm (below the third percentile), and his head circumference was 40 cm (at the 25th percentile). By the age of five months, his weight had increased to 5.05 kg (still below the third percentile). Given these findings and the ongoing health issues, the patient was subsequently transferred to another hospital for further treatment, which included intralipid feeding, total parenteral nutrition (TPN), and specialized pediatric gastrointestinal care.

**Figure 1:**
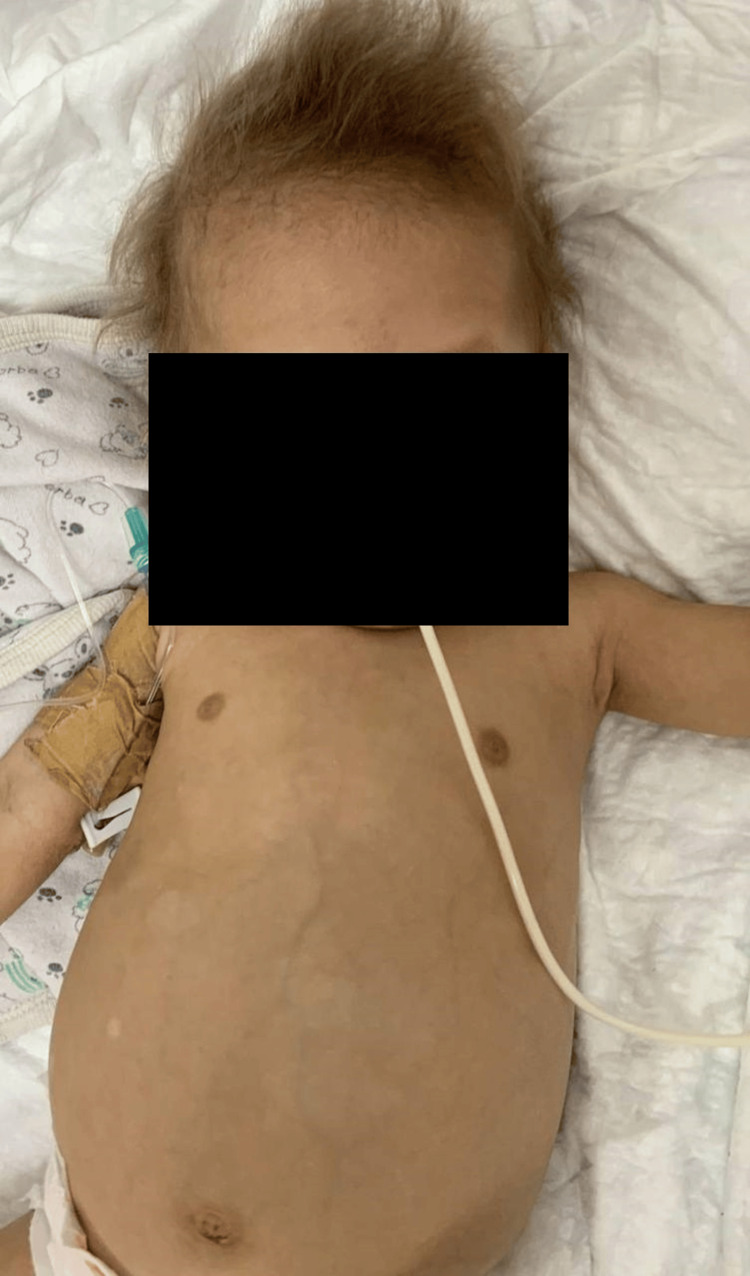
Dysmorphic features associated with THES in the patient at the age of eight months The figure illustrates abdominal skin hypopigmentations and scalp hair abnormalities. THES: tricho-hepato-enteric syndrome

The patient is currently eight months old and remains in the PICU. The patient's weight is currently 5.6 kg (severely below the third percentile). His treatment regimen includes metronidazole, monthly IVIG, broad-spectrum antibiotics, and TPN. Additionally, supplements of iron, zinc, and vitamin D are being administered. The patient's dysmorphic features are illustrated in Figure 1.

## Discussion

THES is a rare and severe bowel disorder that affects the enterocyte epithelium, leading to various histological abnormalities such as clustering enteropathy and a series of microvillus inclusion diseases. It is characterized by multiple key features, including intrauterine growth restriction or being small for gestational age, congenital intractable diarrhea, distinctive hair abnormalities (notably uncombable, brittle hair with features resembling trichorrhexis nodosa), and facial dysmorphism (including a prominent forehead, broad nasal root, and hypertelorism). Skin abnormalities, such as xerosis, "café-au-lait" spots, and angiomas were present in 50% of patients. Additionally, chronic liver disease that can progress to cirrhosis in severe cases is observed. Mild intellectual disability affects about 60% of individuals, along with immunodeficiency characterized by recurrent infections, hypogammaglobulinemia, inadequate vaccine response, and/or elevated levels of immunoglobulin A and/or T-cell production. Less common manifestations may include congenital heart anomalies and platelet abnormalities [1,3,4].

THES is classified into two types: type 1 (associated with *TTC37*), representing 69% of cases, and type 2 (associated with *SKIV2L*), constituting the remaining percentage. Mutation in these genes results in the loss of function of either protein in the Ski complex: *SKIC3* (a TPR-containing protein) or *SKIC2* (an RNA helicase). The Ski complex, a heterotetrameric cytoplasmic cofactor of the RNA exosome, is essential for exosome-mediated RNA surveillance, including the regulation of normal mRNA and the decay of nonfunctional mRNA [5].

Eckard et al. [6] proposed that *SKIC2* and *SKIC3* exhibit distinct and autonomous functions. They noted that cells from individuals with *SKIC2* pathogenic variants (formerly known as SKIV2L) display an interferon 1 signature, whereas cells from individuals with *SKIC3* pathogenic variants (formerly known as TTC37) do not exhibit this signature. Recent research analyzed a cohort of 96 individuals with either *SKIC2* or *SKIC3* mutations and indicated that patients with the *SKIC2* pathogenic variants exhibited more severe symptoms, especially in terms of liver damage and prenatal growth impairment [2].

The current primary management regimen involves parenteral nutrition, enteral feeding, and comprehensive supportive care targeting other affected organs. Early neurorehabilitative therapy should be instituted once the developmental delay is identified. Additionally, in select cases, therapeutic modalities such as corticosteroids, immunosuppressants, and immunoglobulin therapy are employed. Nonetheless, long-term parenteral nutrition may be contraindicated in patients with concomitant liver disease [2,7].

Hepatic manifestations associated with THES may emerge independent of reliance on parenteral nutrition. In a study by Fabre et al. [8], it was observed that among 22 patients diagnosed with THES, 55% presented with liver disease. Furthermore, hepatic cirrhosis was documented in 50% of 18 patients, while hepatic hemosiderosis was noted in 24% of 17 patients, suggesting a potential role of iron overload in the pathogenesis of THES.

In a cohort comprising 15 patients, it was observed that 61.9% of the surviving individuals surpassed the age of 15 years. Within this cohort, the median age at the time of mortality was noted to be 86 months, with mortality primarily attributed to severe infections, malnutrition, and dehydration. Notably, three patients successfully transitioned away from parenteral nutrition. Overall, the prognosis exhibits variability contingent upon the extent of intestinal involvement and the patient's immune status [3].

THES should be considered in the differential diagnosis when an infant presents with persistent diarrhea and failure to thrive since birth, particularly if these symptoms are accompanied by hair and facial abnormalities and immunodeficiency. Despite its rarity as a clinical entity, awareness of THES increased significantly among physicians in recent years [9].

## Conclusions

THES manifests with a diverse range of symptoms, which include persistent diarrhea, unique hair abnormalities, facial dysmorphism, and liver-related symptoms. Genetic testing has identified two subtypes of THES, associated with the genes *SKIC2* and *SKIC3*. The management of this condition can be complex, necessitating a comprehensive, multidisciplinary approach that includes enteral feeding and supportive care, specifically tailored to the needs of the affected organs.
